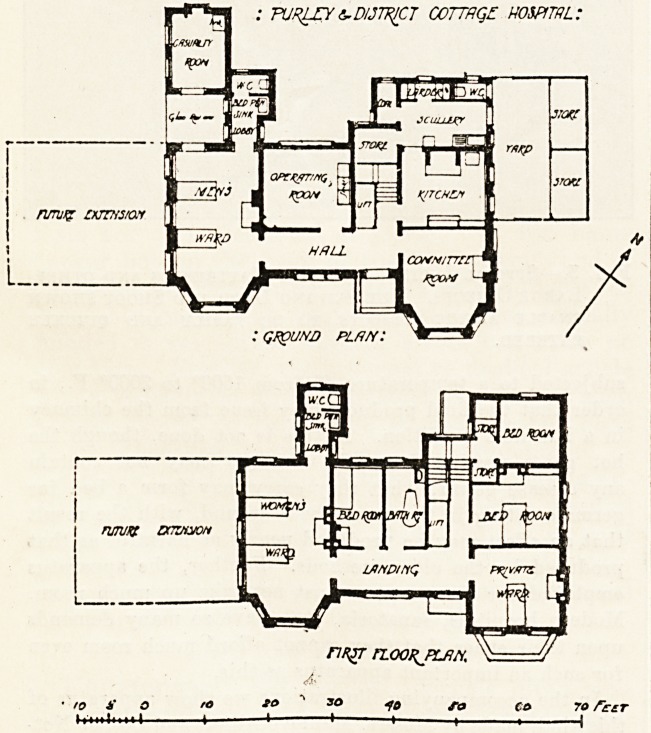# Purley Cottage Hospital

**Published:** 1909-08-14

**Authors:** 


### PURLEY COTTAGE HOSPITAL.

This small hospital was opened by H.R.H. Princess Christian on March 31 last. It is planned at present for five beds on two floors, and provision is made for future extension by which the total number would be increased by some ten beds. This is on the assumption that in each of the wings shown by the dotted lines on the plan, five additional beds could be placed on each floor. No provision seems, however, to be made for any increase in the staff—beyond the three provided for in the plan. A total of three persons, to include both nursing and servant staff, is surely inadequate even for a cottage hospital. The building appears to have been planned on the most rigidly economical lines, there being only one bath-room available for patients and staff alike. No w.c. is provided for nurses, who would either have to use the outside one in common with the servants, or that attached to the female ward.

Apart from these criticisms, the building is planned in a compact and straightforward way, and the elevations are simple and suitable to the purpose.

The hospital was designed by Mr. John Newton, A.R.I.B.A., of Purley.

## Figures and Tables

**Figure f1:**